# Exploring Hydrogenotrophic Methanogenesis: a Genome Scale Metabolic Reconstruction of Methanococcus maripaludis

**DOI:** 10.1128/JB.00571-16

**Published:** 2016-11-18

**Authors:** Matthew A. Richards, Thomas J. Lie, Juan Zhang, Stephen W. Ragsdale, John A. Leigh, Nathan D. Price

**Affiliations:** a Institute for Systems Biology, Seattle, Washington, USA; b Department of Microbiology, University of Washington, Seattle, Washington, USA; c Jiangnan University, Wuxi, China; d Department of Biological Chemistry, University of Michigan Medical School, Ann Arbor, Michigan, USA; University of Tennessee

## Abstract

Hydrogenotrophic methanogenesis occurs in multiple environments, ranging from the intestinal tracts of animals to anaerobic sediments and hot springs. Energy conservation in hydrogenotrophic methanogens was long a mystery; only within the last decade was it reported that net energy conservation for growth depends on electron bifurcation. In this work, we focus on Methanococcus maripaludis, a well-studied hydrogenotrophic marine methanogen. To better understand hydrogenotrophic methanogenesis and compare it with methylotrophic methanogenesis that utilizes oxidative phosphorylation rather than electron bifurcation, we have built iMR539, a genome scale metabolic reconstruction that accounts for 539 of the 1,722 protein-coding genes of M. maripaludis strain S2. Our reconstructed metabolic network uses recent literature to not only represent the central electron bifurcation reaction but also incorporate vital biosynthesis and assimilation pathways, including unique cofactor and coenzyme syntheses. We show that our model accurately predicts experimental growth and gene knockout data, with 93% accuracy and a Matthews correlation coefficient of 0.78. Furthermore, we use our metabolic network reconstruction to probe the implications of electron bifurcation by showing its essentiality, as well as investigating the infeasibility of aceticlastic methanogenesis in the network. Additionally, we demonstrate a method of applying thermodynamic constraints to a metabolic model to quickly estimate overall free-energy changes between what comes in and out of the cell. Finally, we describe a novel reconstruction-specific computational toolbox we created to improve usability. Together, our results provide a computational network for exploring hydrogenotrophic methanogenesis and confirm the importance of electron bifurcation in this process.

**IMPORTANCE** Understanding and applying hydrogenotrophic methanogenesis is a promising avenue for developing new bioenergy technologies around methane gas. Although a significant portion of biological methane is generated through this environmentally ubiquitous pathway, existing methanogen models portray the more traditional energy conservation mechanisms that are found in other methanogens. We have constructed a genome scale metabolic network of Methanococcus maripaludis that explicitly accounts for all major reactions involved in hydrogenotrophic methanogenesis. Our reconstruction demonstrates the importance of electron bifurcation in central metabolism, providing both a window into hydrogenotrophic methanogenesis and a hypothesis-generating platform to fuel metabolic engineering efforts.

## INTRODUCTION

Biologically produced methane is a topic of significant interest based on both environmental impacts and bioenergy uses. Methane is produced in the environment by biological and nonbiological sources (1) and plays a critical role in the global carbon cycle. For example, a large proportion of anaerobic biomass metabolism is coupled to methanogenesis, which is responsible for the annual generation of 1 Gt of methane in the biosphere (2). Methane is also the second most abundant greenhouse gas after carbon dioxide (3) and is 21 times more potent than CO_2_ (4) in absorbing and emitting energy. In terms of its role in bioenergy, methane is the major component (∼87%) of natural gas, used as a heating fuel in 22% of U.S. homes. It is also a candidate bridge fuel (5), an energy source that aids the transition from traditional fossil fuels to fully renewable sources, because it produces more heat per mass unit (55.7 kJ/g) than any other hydrocarbon, plugs into a substantial existing infrastructure, and burns comparatively cleaner than traditional fossil fuels. Advancing technology also enables this gas to be converted to high-energy-density liquid fuels with a lower carbon footprint (6).

Methanogenic Archaea grow on carbon dioxide or simple carbon compounds and produce methane as a metabolic waste product. The environmental ubiquity of this microbial group makes it the largest biological contributor of methane on this planet. Although phylogenetically and metabolically diverse, methanogens can be separated into two groups based on the presence or absence of cytochromes (2). The cytochrome-lacking methanogens (sometimes referred to as hydrogenotrophic methanogens) mainly use H_2_, and sometimes formate, as sources of electrons for CO_2_ reduction to methane. In contrast, cytochrome-containing (or methylotrophic) methanogens utilize acetate and methylated compounds for methanogenic growth, with a minority also being able to use H_2_ and CO_2_. Although both groups have similar central pathways of CO_2_ reduction, there are also differences in energy conservation (7) at the last methanogenic step involving heterodisulfide reductase (Hdr).

The reduction of the CoM-S-S-CoB heterodisulfide (where CoM and CoB are coenzyme M and coenzyme B, respectively) with H_2_ or reduced electron carriers is exergonic and can be directly or indirectly coupled to energy generation. In the methylotrophic methanogens, a membrane-associated cytochrome-containing Hdr (HdrDE) receives reducing equivalents from a methanogen-specific membrane-soluble electron shuttle, methanophenazine, for reduction of the heterodisulfide. This results in proton extrusion and the creation of a membrane potential for ATP generation (8, 9). However, in the hydrogenotrophic methanogens, the Hdr (HdrABC) contains flavin instead of heme, is cytoplasmic, and generates no membrane potential. Instead, Hdr mediates a bifurcation of electron flow (likely via the flavin group [10]) in which the exergonic heterodisulfide reduction is coupled to and drives the endergonic reduction of a ferredoxin used for the first step of methanogenesis (11).

Methanococcus maripaludis (12) belongs to the group of hydrogenotrophic cytochrome-lacking methanogens. Compared to the larger genomes of methylotrophic methanogens, its genome is relatively small and contains only 1,722 protein-coding genes (13). It grows robustly, with a doubling time of 2 h (12), and is genetically tractable (14); thus, it has been an ideal candidate for studying methanogenesis, unique cofactors and their biosynthesis (15), and gene regulation (16). To avoid environmental fluctuations that can affect gene regulation, a system for continuous culture of M. maripaludis (17) has been established for steady-state transcriptomic (18) and proteomic (19) studies of M. maripaludis strains. Several groups have also employed larger systems biology approaches to perform predictive studies using this organism (20). With these tools in place and the ability for expression of heterologous genes in M. maripaludis (21, 22), the metabolic engineering of M. maripaludis for industrial use is a clear next step.

Genome scale metabolic reconstructions are powerful tools that map and elucidate metabolic pathways. They are organism-specific knowledge bases that can be used for simulating steady-state growth via flux balance analysis (FBA) (23) by generating constraint-based models. Using these models, we can hypothesize different metabolic scenarios that can then be tested experimentally. They have helped guide metabolic engineering efforts to produce industrial biochemicals in multiple organisms (24, 25). Similarly, a genome scale metabolic reconstruction for M. maripaludis would not only promote a better understanding of methanogenesis but also support metabolic engineering efforts that could harness the unique metabolism of this hydrogenotrophic methanogen. Other groups have already created metabolic models of M. maripaludis as part of a mutualistic community model with Desulfovibrio vulgaris (26) and under axenic conditions (27). In the former case, the model of M. maripaludis included 82 reactions and 72 intracellular metabolites that represented only core metabolism and was used primarily to investigate interactions between the two different species rather than map out more comprehensively the organism's metabolism (26). The latter case was the first genome scale metabolic reconstruction of M. maripaludis (27), an important step toward understanding M. maripaludis metabolism. However, that model relied heavily on the KEGG and MetaCyc databases, utilizing only 16 additional literature sources, a small amount of reference materials that resulted in the omission of many important biosynthetic pathways. In addition, the model erroneously utilized methanophenazine in an HdrDE-dependent electron transport chain and omitted electron bifurcation. Much work remains to fully map this complex network and better represent biochemically characterized pathways through close integration of experimental and computational efforts.

In this genome scale metabolic reconstruction, iMR539, we include 539 genes and 688 metabolic reactions spanning the vital catabolic and biosynthetic pathways important in the metabolism of M. maripaludis. We describe important updates, corrections, and refinements, based on recent literature, to the previous metabolic models. The most critical addition is the electron bifurcation step, which explains the ability of this organism to grow despite the lack of a proton-exporting electron transport chain. This correction also eliminated methanophenazine utilization and synthesis, which is part of the membrane-bound electron transport system of the methylotrophic methanogens and is absent from hydrogenotrophic methanogens (2). Additional features include a corrected sulfur assimilation pathway (28) and the addition of biosynthesis pathways for all of the unique coenzymes involved in methanogenesis (15). We increased genome coverage by employing likelihood-based gap filling, a recently developed technique that fills reaction gaps based on gene homology rather than on parsimony (29). Furthermore, we expanded the scope of our reconstruction beyond stoichiometric considerations by creating a method to approximate overall model free energy. This is an especially salient consideration for methanogenic archaea, which can grow close to the thermodynamic limits that support life (30). A well-established method of applying free-energy constraints involves applying the second law of thermodynamics to metabolic models to restrict reaction directionalities in the direction of negative free-energy generation (31, 32). Rather than apply thermodynamic constraints to every metabolic reaction as in the aforementioned approach, we created a simple flux-balance accounting to estimate overall free-energy change during steady-state growth based solely on standard free energies and effective concentrations of external metabolites. In combining these thermodynamic considerations with stoichiometric information, iMR539 provides a means to predict energetically feasible strain designs, enhancing our metabolic engineering capabilities with M. maripaludis. Using our reconstruction, we tested the essentiality of bifurcation for hydrogenotrophic methanogenesis, investigated the inability of M. maripaludis to grow on acetate as a carbon and energy source, and predicted growth phenotypes for hydrogenase mutants.

## MATERIALS AND METHODS

### Genome scale reconstruction procedure.

The process of genome scale metabolic network reconstruction has been reviewed previously (33) and begins with annotating an organism genome using gene-protein-reaction (GPR) relationships stored in a reaction database. Several databases are available for this purpose (34–36); we chose the Department of Energy Systems Biology Knowledgebase (Kbase; www.kbase.us), a suite of tools that includes the Model SEED reaction database (36). We created our first draft reconstruction using the stored Kbase genome for M. maripaludis S2 (genome identifier kb|g.575) and the automated reconstruction method (“reconstruct genome scale metabolic model”). For this initial reconstruction, we used the default Gram-negative biomass composition and filled knowledge gaps using likelihood-based gap filling (this method currently is not supported in the Kbase Narrative Interface). This yielded a first full draft of the metabolic reconstruction that could predict growth when simulated as a model.

We expanded and refined the model by manually adding information from literature sources. In cases where reactions from literature were part of the Model SEED database, we labeled the reactions using SEED identifiers, names, subsystems, and EC numbers. For other cases where we encountered reactions that were not part of the Model SEED, we created unique reaction identifiers and names and then added subsystem information based on our knowledge of the metabolic network. We also adhered to SEED identifiers, names, formulas, and charges for metabolites whenever possible and had very few cases where we specified our own values. Metabolites were compartmentalized using standard tags for cytosol (“c0”) and extracellular (“e0”) compartments. These tags additionally identify M. maripaludis as “Organism 0” in the possible future case where we could add other organisms to create a community metabolic reconstruction. Exchange reactions used for introducing metabolites to the extracellular compartment were standardized in “EX_{metabolite ID}[e0]” format. Comprehensive information on the reactions, metabolites, and genes in our reconstruction can be found in Data set S1 in the supplemental material.

### Model simulations with flux balance analysis.

To make rigorous quantitative growth predictions, a genome scale metabolic reconstruction can be simulated as a model. Reactions and their participating metabolites in the metabolic network are connected via the stoichiometric matrix (*S*), which contains the stoichiometric coefficients for each metabolite (row) in each reaction (column). The *S* matrix is used as the basis of a model via the principles of metabolite mass conservation by recognizing that time-dependent accumulation of metabolites in the system (*b*) is equivalent to the product of the *S* matrix and the vector of reaction fluxes (*v*):

Sv=b

In flux balance analysis (FBA), we further simplify this differential system by assuming our organism is in steady-state growth; thus, *b* equals 0 and the system is linear (37). This assumption bounds our model system to a large solution space that can be constrained further by applying upper and lower bounds to each individual reaction flux (*v_i_*):

$$v_{i,\;\text{lower}}\leq v_{i}\leq v_{i,\;\text{upper}}$$

To find feasible flux distributions that represent likely physiological states within this solution space, we solved our model by optimizing the biomass objective function, a simulation of maximum cell growth yield (38). Mathematically, this is represented as the product of the reaction fluxes and the objective vector (*c*), which in this case is 0 for all indices except biomass production:

Maximize c′v

We further constrained possible flux distributions by minimizing the squared sum of fluxes (minNorm = “one” in call to optimizeCbModel.m), effectively forcing our model to find solutions that minimize the total flux in the system while maximizing growth:

$$Minimize\sum_{i}^{m}v_{i}^{2}$$

Adding this optional parameter cut down on reactions with needlessly high fluxes, which typically results in more realistic flux predictions. All model simulations were performed using the COBRA Toolbox 2.0 (39) in MATLAB (7.14.0.739) (The MathWorks Inc., Natick, MA).

To encourage model transparency (40) and assist future users in simulating condition-specific models, we designed several functions that create these models, simulate maximum growth with the aforementioned constraints, and print relevant information from the flux distribution (see Text S4 in the supplemental material). We also wrote numerous functions to help modify the reaction network, retrieve specific useful pieces of information from model simulations, and diagnose issues that may arise during model use. For several of these functions, we used the Paint4Net toolbox (41) to draw flux maps that show the direction and magnitude of fluxes in a given FBA solution. A description of some of our functions in their current versions is included (see Text S4) with the full set of up-to-date tools available on GitHub (https://github.com/marichards/methanococcus).

### Gene knockout phenotype simulations.

Because a model is based around the stoichiometry of reactions contained in the *S* matrix, knocking out a gene is akin to knocking out all reactions that depend on the gene. Thus, performing a gene knockout phenotype simulation in a metabolic model requires that model reactions be linked to genes via GPR relationships. We performed gene knockout simulations using our function “simulateKOPanel.m” (see Text S4 in the supplemental material), which relies heavily on the “deleteModelGenes.m” function in the COBRA Toolbox 2.0 (39) as well as several of our own functions. Our experimental test set was comprised of 30 total experimentally verified gene knockouts, including 18 unique genotypes and 4 unique growth conditions (42–47). We simulated growth phenotypes for all 30 of these wet-laboratory experiments as well as the 42 other possible genotype-medium combinations that did not correspond to experimental data. For all 72 combinations of knockout genotypes and growth conditions, we evaluated the predicted growth phenotypes as lethal/nonlethal with a threshold of 10% wild-type growth. Predictive accuracy was assessed by comparing predictions on the 30 known phenotypes with wet-laboratory data; the remaining 42 predictions had no associated wet-laboratory data for validation and could not be assessed for accuracy. We further evaluated our model's performance using the Matthews correlation coefficient (MCC), a metric that evaluates correlation based on a −1 to 1 scale (48):

$$\text{MCC}=\frac{\left(\text{TP}\times \text{TN})-(\text{FP}\times \text{FN}\right)}{\sqrt{\left(\text{TP}+\text{FP})(\text{TP}+\text{FN})(\text{TN}+\text{FP})(\text{TN}+\text{FN}\right)}}$$

where TP is true positive, TN is true negative, FP is false positive, and FN is false negative. We interpreted nonlethal gene knockouts as positive growth and lethal gene knockouts as negative growth.

### Thermodynamic calculations.

When simulating optimal growth using a metabolic model, we expect that our system must necessarily have negative overall free energy to support growth. We added standard free energies of formation (1 mM, 25°C, 10^5^ Pa, pH 7, ionic strength of 0.1 M) from the Equilibrator database (49) to all exchange reactions for which these values could be reliably estimated via the group contribution method (50). These exchanges effectively represent the organism's overall biochemical reaction; therefore, it is reasonable to expect this overall reaction must produce a negative overall free energy to support growth. To incorporate these values into our reconstruction, we expanded the standard model structure to include a “freeEnergy” numerical array with length equal to that of the “reactions” array. For calculating overall free energy of a flux distribution, we created an “optimizeThermoModel.m” function (see Text S4 in the supplemental material) that is built around the “optimizeCbModel.m” function in the COBRA Toolbox 2.0 (39). Our script accepts effective concentrations (millimolar) for specified exchange metabolites, assumes standard activities of 1 mM for unspecified metabolites, and uses these values to calculate effective metabolite free energies based on the reconstruction's stored values for each exchange reaction. Prior to performing FBA, we add these free energies to the exchange reactions, which ordinarily have the form

$$A_{\text{e}0}\;\text{⇌}$$

We alter these exchanges such that production of a metabolite creates free energy equivalent to the metabolite's free energy of formation:

$$A_{\text{e}0}\;\text{⇌}(\Delta G_{A\text{e}0})\text{dG}$$

where Δ*G_A_*_e0_ is the stoichiometric coefficient of a new metabolite, dG, that is used to sum model free energy. Because exchange reactions must satisfy mass balance by necessarily entering or exiting the model without creating new metabolites, adding free energies to the model creates an imbalance that we must correct. We restore model balance by allowing dG to exit the model via its own exchange reaction (GIBBS_kJ_GDW):

dG ⇌

Measuring the total flux of the exchange reaction gives an estimation of total free energy being generated in an FBA solution on a per-cell-mass basis. We have incorporated this thermodynamic calculation into all of our available model simulations (see Text S4 in the supplemental material); thus, by default, we calculate and print overall model free energy in every flux distribution. Optionally, this calculation can be used as an additional model constraint that restricts overall free energy to be negative, the equivalent of imposing the second law of thermodynamics on the organism itself. For an example that demonstrates this method for predicting overall free energy over a range of H_2_ levels, see Text S3.

### Dry cell weight and growth yield measurements.

Wild-type M. maripaludis S2 cells were grown in McNA medium, a chemically defined medium for growth on H_2_ and CO_2_ supplemented with acetate (see Data set S1 in the supplemental material), using a 1-liter chemostat under anaerobic conditions as described previously (17). The chemostat was operated in steady-state continuous mode under H_2_-limiting conditions to match model simulation conditions, with gas flows of 10 to 20 ml/min H_2_, 40 ml/min CO_2_, 15 ml/min of an H_2_S-Ar mixture (1:99, vol/vol), and a balance of N_2_ up to a total of 200 ml/min. We altered our growth rate of M. maripaludis by varying pump speeds to achieve dilution rates of approximately 0.045 to 0.090 h^−1^, checking the optical density at 660 nm (OD_660_) periodically to ensure steady state at each data point. For each sample point, we measured growth rate based on dilution rate and methane evolution rate via a combination of a bubble flow meter to assess total gas outflow and a Buck Scientific model 910 gas chromatograph equipped with a flame ionization detector to quantify the methane fraction.

We recalculated calibration curves for dry cell weight versus optical density by measuring dry cell weight via cell filtering and OD_660_ via a UV-visible spectrophotometer (path length of 13 mm; Spectronic 20D+) blanked with water. After measuring chemostat optical density, we sampled 50-ml aliquots of cells in suspension directly from chemostat culture and centrifuged samples at 8,656 × *g* (7,000 rpm) for 15 min. Forty milliliters of supernatant was removed by pipette, and then cells were resuspended in the remaining 10 ml of medium. These concentrated aliquots were vacuum filtered through 0.45-μm-pore filters to remove all noncellular components, dried at room temperature, and weighed daily until their weights stabilized.

Growth yields (*Y*; in grams of dry weight per mole of CH_4_) were calculated based on doubling time [*t_d_*; equal to ln(2) × (dilution rate × 60)^−1^] as described previously (51) but with our measured conversion between OD_660_ and cell density:

$$Y=\frac{{\text{OD}}_{660}}{{\text{CH}}_{4}}\times \frac{0.46\;\text{g}/\text{liter}}{\text{O}{\text{D}}_{600}}\times \frac{1}{t_{d}}\times \frac{22,400\text{ ml}}{\text{mol}}$$

where CH_4_ is measured in milliliters per minute and *t_d_* is in minutes.

### ATP maintenance and predicted growth yields.

As described by Thiele and Palsson, the optimal way to obtain accurate ATP maintenance values is to plot ATP production versus growth data from chemostat growth experiments (33). In practice, this requires measuring steady-state growth rate in concert with an uptake rate or, in our case, a product secretion rate, as described above. To calculate ATP maintenance values in our model, we constrained our model to our measured growth rate and methane secretion rate at each sampling point and set the model objective to maximize ATP hydrolysis (rxn00062[c0]). We plotted each resulting value of ATP production as a function of growth rate and obtained the growth-associated (slope) and non-growth-associated (*y* intercept) ATP maintenance values using a linear model, as described by Thiele and Palsson (33). The resulting plot can be found in Text S2 in the supplemental material.

Our growth data points comprised a set of 9 measurements. To mitigate overfitting issues, we employed leave-one-out cross validation (LOOCV) in estimating and then testing effects of ATP maintenance estimation. In the LOOCV approach, a set of *n* samples was divided into a training data set of *n* − 1 points and a test sample of 1 point. The model developed on the training set was then tested on the remaining point that was left out of the training data. In employing this method for each of our 9 measurements, we determined ATP maintenance values for the *n* − 1 data set as described above to create a trained model. We then constrained our model's methane secretion flux to the measured rate in the remaining test point and predicted maximum growth rate within that constraint using our trained model. Using these values, we calculated predicted growth yields for each point using the above-described formula and compared them to our measured values for each point. All simulations were performed using the default H_2_ plus CO_2_ medium formulation supplemented with acetate (McNA medium).

### Reconstruction and model availability.

Reconstructing a metabolic network is an iterative process; therefore, to encourage future updates and expansions, it is paramount that reconstructions be as clear as possible (40). We have strived for clarity in both our nomenclature and in our decision-making process for including each reaction present in our reconstruction. Reactions and metabolites in our network are based upon identifiers and names found in Kbase but also include cross-links to ChEBI (52) and KEGG identifiers (34), enzyme commission numbers, and reaction subsystems where available. Each reaction in the reconstruction is also connected to its literature and/or database source, plus its reaction confidence score when applicable (see Data set S1 in the supplemental material).

Additionally, we have sought to maximize usability of both our reconstruction and our model. The systems biology markup language (SBML) is a standard medium for distributing metabolic reconstructions (53); thus, we have included our reaction network in SBML level 2, the highest version currently supported by the COBRA Toolbox (39). In our experience using reconstructions from other groups, we have found a wide range of usability, from those that can easily be imported and simulated to those that are difficult to use and interpret. In the interest of making our simulations and results easy to reproduce, we have included our reconstruction in MATLAB data structure format and all of our scripts for simulating model growth on different media and gene knockout phenotypes (see Text S4 in the supplemental material).We have made our scripts and reconstruction available on GitHub (https://github.com/marichards/methanococcus) and have deposited our SBML model in the BioModels Database (54) with the identifier MODEL1607200000.

## RESULTS

### Basic reconstruction statistics.

The basic statistics for iMR539 are displayed in Table 1. Notably, reactions are categorized as (i) internal reactions, occurring entirely within the cytoplasm; (ii) transport reactions, involving translocation of at least one chemical species across the cell membrane; and (iii) exchange reactions, which supply metabolites to or remove metabolites from the model. Of the 580 internal reactions in our network, 85.7% have been assigned to at least one gene. This is a rather high percentage, eclipsing that of the previous M. maripaludis reconstruction (81.4%) (27) and comparing favorably to reconstructions of fellow methanogens Methanosarcina barkeri and Methanosarcina acetivorans (85.7% and 85.1%, respectively) (55, 56). We suspect that a major reason for this high percentage of gene-associated reactions was our use of likelihood-based gap filling (29), which resulted in the automated addition of 66 genes to our reconstruction before manual curation. Furthermore, we relied heavily on biochemical knowledge from literature sources, particularly regarding recently elucidated biosynthesis pathways that were not correctly annotated in annotation databases. Our combined use of maximum-likelihood gap filling and reliance on published literature sources are the likely explanations for our consistent ties to gene homology.

**TABLE 1:** General statistics for the iMR539 reconstruction^*a*^

Parameter^*a*^	Value(s)
Protein-encoding genes (*n*)	539
% ORF coverage	31
Intracellular/extracellular metabolites (*n*)	657/53
Dead-end metabolites (*n*)	260
Internal reactions (*n*)	580
Transport/exchange reactions (*n*)	49/59
Dead-end reactions (*n*)	206
Gene-associated reactions (*n*)	497

a M. maripaludis S2 model statistics. ORF, open reading frame.

Another salient detail of our reconstruction is that it includes many dead-end metabolites and reactions that cannot be synthesized or consumed. Although such metabolites and reactions cannot yet be included in our simulatable model, because they all have at least one gene association supporting their involvement in metabolism, we have included them in our metabolic reconstruction. They represent excellent candidates for further exploration of M. maripaludis metabolism, particularly as full synthesis or consumption pathways are elucidated, allowing iMR539 to be updated and expanded in the future.

**FIG 1:**
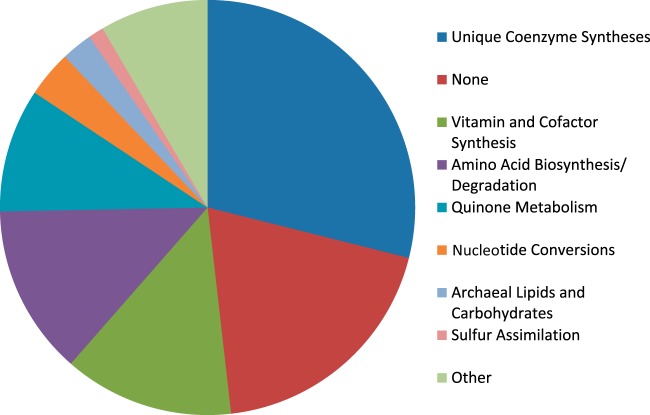
Chart showing broad subsystem groupings of the 83 reactions in iMR539 that are not associated with any genes. Reactions falling underneath the “None” subsystem grouping were present in the Model SEED database but had no subsystems listed there and no obvious membership in another subsystem. Reactions grouped within “Other” were dissimilar both from the other categories and from one another; thus, we felt they did not merit creation of multiple additional categories.

Conversely, our reconstruction contains 83 internal reactions that lack genes, many of which were added during automated gap filling but some of which were added manually. All of our reactions are annotated with subsystems, allowing us to assess where each reaction (including those without genes) fits into metabolism. Figure 1 shows the breakdown of reactions without genes, where the subsystems have been manually grouped into broader categories (e.g., “Amino Acid Biosynthesis” instead of “Glycine Biosynthesis”). The largest group of these reactions is “Unique Coenzyme Syntheses,” which includes reactions that synthesize coenzyme M, coenzyme B, tetrahydromethanopterin (H_4_MPT), methanofuran, coenzyme F_420_, and coenzyme F_430_. Although these 24 reactions lack genes, all of them were added manually as hypothetical steps to complete essential biosynthetic pathways and are based on information from biochemical literature. These are distinct from the 11 reactions encompassed by “Vitamin and Cofactor Synthesis” that were added to fill biosynthesis gaps but have no supporting literature evidence. We expect that as experimental research groups uncover more biochemical phenomena, they will determine genes that are tied to the reactions in the “Unique Coenzyme Synthesis” group. These gap-filling reactions, much like dead-end reactions and metabolites, point toward poorly understood areas of metabolism that require more investigation into both the reaction pathways and their associated genes.

As an additional feature of our reconstruction, our use of likelihood-based gap filling also assigned likelihood scores for many of the reactions in the reconstruction. These confidence scores quantify the probability of a given reaction being part of the metabolic reconstruction on a scale of 0 to 1 and provide a new metric of evaluating our confidence in the reconstruction (see Data set S1 in the supplemental material). We can then use the scores both to quickly hone in on reactions that lack genes and gene-associated reactions with low gene homology as possible targets for future experimental investigations and to expand upon and improve the existing reconstruction.

### Model prediction of electron bifurcation essentiality in hydrogenotrophic methanogenesis.

**FIG 2:**
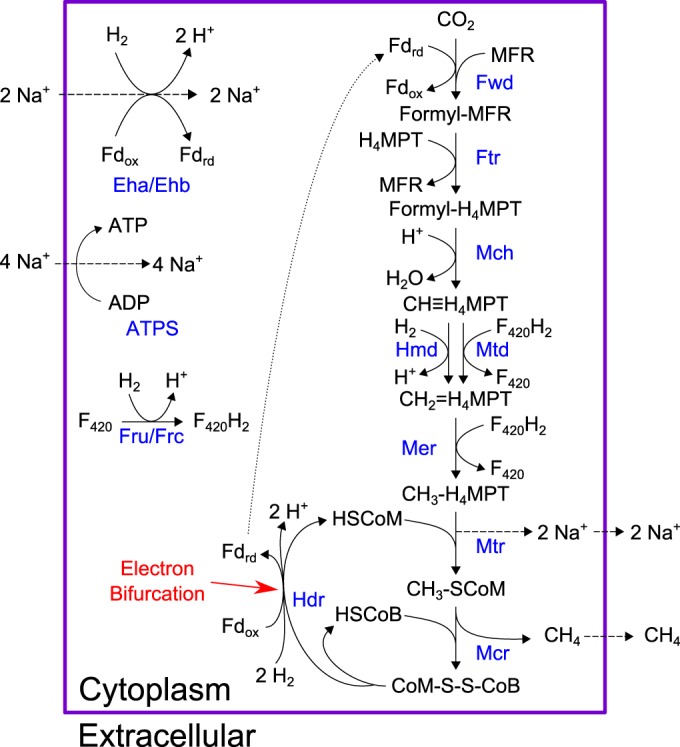
Native pathway of hydrogenotrophic methanogenesis present in M. maripaludis. As shown, electrons from 2 mol H_2_ are split between reducing ferredoxin and regenerating coenzymes B and M. Reduced ferredoxin from this reaction links it to CO_2_ reduction, the first step in the pathway. Enzyme names are shown in blue. Metabolites: Fd_rd_, reduced ferredoxin; Fd_ox_, oxidized ferredoxin; MFR, methanofuran; HSCoM, coenzyme M; HSCoB, coenzyme B. Enzymes: Fwd, formylmethanofuran dehydrogenase; Ftr, formylmethanofuran-H_4_MPT formyl transferase; Mch, methenyl-H_4_MPT cyclohydrolase; Hmd, H_2_-dependent methylene-H_4_MPT dehydrogenase; Mtd, F_420_-dependent methylene-H_4_MPT dehydrogenase; Mer, methylene-H_4_MPT reductase; Mtr, methyl-H_4_MPT coenzyme M methyltransferase; Mcr, methyl coenzyme M reductase; Hdr, heterodisulfide reductase; Eha/Ehb, energy-conserving hydrogenases; ATPS, ATP-synthase; Fru, F_420_-reducing hydrogenase (selenocysteine containing); Frc, F_420_-reducing hydrogenase (cysteine containing).

Methanogenesis from H_2_ and CO_2_ is often represented as a linear pathway with heterodisulfide reduction as the final step (Fig. 2). Our model for a hydrogenotrophic methanogen links the heterodisulfide reductase step to the first CO_2_ reduction step via a ferredoxin (2, 43) as described previously, resulting in a cyclical methanogenic pathway (57), CoB − S − S − CoM + 2H_2_ + Fd_ox_ ⇌ HS − + HS − CoM + 2H^+^ + Fd_red_ (reaction 1), where Fd_ox_ is oxidized ferredoxin and Fd_red_ is reduced ferredoxin.

To demonstrate that the linear pathway cannot support growth of M. maripaludis in the absence of the methanophenazine-dependent HdrDE complex, we altered the native electron-bifurcating HdrABC reaction (reaction 1). We removed electron bifurcation from this reaction by removing ferredoxin, balancing mass and charge to yield an altered reaction, CoB − S − S − CoM + H_2_ ⇌ HS − CoB + HS − CoM (reaction 2).

This scenario represented a hypothetical case where M. maripaludis does not contain a membrane-bound HdrDE complex but cannot perform electron bifurcation. Unsurprisingly, we were unable to predict *in silico* growth on CO_2_ plus H_2_. Ferredoxin reduction via electron bifurcation is an essential part of our network; without this energy-coupling step, M. maripaludis would not grow. The alternative source of reduced ferredoxin is the energy-converting Eha hydrogenase, which utilizes a sodium ion gradient to reduce ferredoxin with H_2_ at a 1:1 ratio. CO_2_ reduction to methane requires reduced ferredoxin and pumps out sodium ions, also at a 1:1 ratio. Thus, each cycle of methanogenesis in this scenario effectively produces no net sodium ion gradient for synthesizing ATP, the central component necessary for biomass formation. Overall, this simple exercise illustrates the essentiality of ferredoxin reduction via electron bifurcation and reinforces the idea that Eha hydrogenase can play only an anaplerotic role in methanogenesis (43).

**FIG 3:**
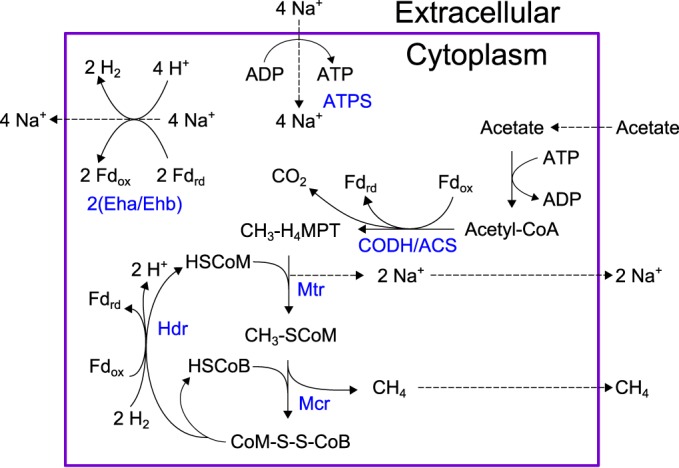
Hypothetical pathway for aceticlastic methanogenesis in M. maripaludis. As demonstrated, this scheme would require 2 cycles of Eha/Ehb in order to oxidize ferredoxin reduced by the CODH/ACS and Hdr reactions. By constraining the Eha/Ehb reaction to only 10% of methane efflux, this pathway becomes infeasible. Enzyme names are shown in blue. Metabolites: Fd_rd_, reduced ferredoxin; Fd_ox_, oxidized ferredoxin; MFR, methanofuran; HSCoM, coenzyme M; HSCoB, coenzyme B. Enzymes: CODH/ACS, carbon monoxide dehydrogenase/acetyl-CoA synthase complex; Mtr, methyl-H_4_MPT coenzyme M methyltransferase; Mcr, methyl coenzyme M reductase; Hdr, heterodisulfide reductase; Eha/Ehb, energy-conserving hydrogenases; ATPS, ATP-synthase.

M. maripaludis can assimilate acetate as a source of carbon, but it cannot replace H_2_ and CO_2_ as an energy source (58). This contrasts with the situation in methylotrophic methanogens, such as Methanosarcina barkeri, that can subsist using solely the aceticlastic pathway (59). It is unknown why M. maripaludis cannot grow on acetate as a source of energy, and our reconstruction did not reveal any strictly stoichiometric obstacle to growth. However, much like the pathway in M. barkeri, an aceticlastic pathway in M. maripaludis would require energy-converting hydrogenases (Eha and Ehb) to produce H_2_ using reduced ferredoxin, pumping out sodium ions, and thrusting this reaction into a central stoichiometric role rather than an anaplerotic one. When we simulated our model and allowed Eha/Ehb unlimited flux, we could predict aceticlastic growth, with Eha/Ehb oxidizing approximately 2 mol of ferredoxin per mol of methane produced (Fig. 3). We then constrained our model to enforce a solely anaplerotic or biosynthetic role of energy-converting hydrogenase by limiting flux through the Eha/Ehb reaction to 10% of the methane secretion rate. Doing so prevented our model from predicting growth from acetate alone but did not restrict hydrogenotrophic growth or supplementary acetate uptake. This simulation supports the hypothesis that M. maripaludis cannot achieve aceticlastic growth because Eha or Ehb cannot assume a central role in methanogenesis. In keeping with these results, we have restricted flux through Eha/Ehb in our model to ≤10% of methane secretion as a default constraint.

### Improvements to the reconstruction of other biochemical pathways.

A major part of our manual curation was adding biosynthetic pathways for the methanogenic coenzymes, sugars, and lipids. M. maripaludis utilizes a number of unusual coenzymes (methanofuran, H_4_MPT, coenzyme F_420_, coenzyme B, coenzyme M, and coenzyme F_430_) as carbon and electron carriers during methanogenesis (60). It also contains recently characterized pathways for synthesizing a tetrasaccharide for N-linked glycosylation of archaellin (archaeal flagellin) (61) and multiple forms of archaeol, an archaeal membrane ether lipid (62). None of these pathways were included in our draft reconstruction, and few were completely present in the Model SEED database (63); thus, the bulk of these reactions were added manually. These biosynthetic pathways, particularly for coenzymes, are required biomass components of M. maripaludis metabolism that set it apart from the vast majority of known biochemistry and are crucial for distinguishing our reconstruction from existing networks.

In a similar vein, we sought to accurately represent sulfur assimilation, a pathway not yet fully understood in M. maripaludis. Sulfate is known not to be the sulfur source for M. maripaludis; moreover, sulfate reduction would produce sulfite, a methanogenesis inhibitor (64). However, because sulfate is the default sulfur source for most microorganisms, our first draft reconstruction included a sulfate transporter and sulfate reduction pathway. We removed this default pathway and instead added a pathway to utilize hydrogen sulfide gas, the primary sulfur source for M. maripaludis (65). Our updated sulfur assimilation pathway includes sulfide oxidation to sulfite, an essential metabolite for multiple biosynthetic pathways, via a hypothesized dissimilatory sulfite reductase-like protein (28).

### Growth yield validation and ATP maintenance.

**FIG 4:**
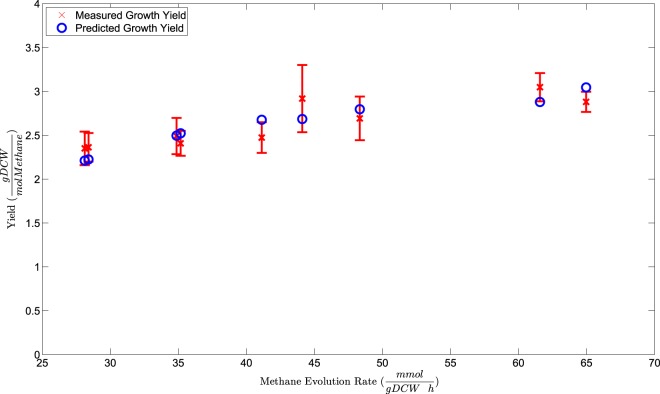
Comparing growth yield predictions on hydrogen to measured data using LOOCV (see Materials and Methods). All but two predicted growth rates fall within the 95% confidence interval of the measured values. Both of the outlying predictions are for slightly higher growth yields than were measured. gDCW, grams of dry cell weight.

Evaluating a metabolic network reconstruction by qualitatively comparing it to known biochemical phenomena is a valuable way to gauge how close the network can represent actual biochemistry. To make more quantitative comparisons, we must convert the reconstruction to a metabolic model by imposing flux constraints on the network, enforcing mass balance on all metabolites, and optimizing to an objective function (see Materials and Methods). A standard way to quantitatively evaluate the resulting model is to simulate maximum cell growth under steady-state conditions and compare growth yield predictions to experimentally determined values. There is scarce published growth yield data for M. maripaludis; thus, we generated our own experimental growth yield measurements. We conducted chemostat growth experiments under H_2_-limiting conditions and measured growth yields as described previously (51), but we varied our dilution rate to gather a range of different yield measurements. Cell density was assessed as the OD_660_. Previous measurements at 600 nm determined a conversion factor of an OD_600_ of 1, corresponding to 0.34 mg (dry weight) · ml^−1^ (44). Using a combination of centrifugation and vacuum filtering (see Materials and Methods), we plotted a new calibration curve (see Text S2 in the supplemental material) and determined that an OD_660_ of 1 corresponds to 0.462 ± 0.015 mg (dry weight) · ml^−1^. Using this value, we determined growth yields and growth rates (equal to dilution rates) and compared them to measured methane evolution rates (see Materials and Methods). Measured growth yields for nine independent steady-state time points are plotted in Fig. 4.

We then tested our model by generating growth yield predictions and comparing them to measured growth yields. Growth yield predictions depend both on metabolic steps, where ATP is generated or hydrolyzed, and on ATP maintenance energies (55). From a modeling perspective, maintenance energies include the moles of ATP needed to support cellular processes not otherwise depicted in metabolism, including DNA replication, RNA transcription, protein synthesis, and other requirements. We recognized that our model was essentially untrained in terms of ATP maintenance and contained automated values from our first draft reconstruction. Thus, it was crucial to train our model by fitting it to our experimental data set. However, we were also wary of overfitting our model by training and testing on the same set of samples. We addressed both concerns by performing LOOCV on our full data set. Thus, for each of our nine growth rate values, we used the remaining eight growth rates and their associated measured methane evolution rates to derive ATP maintenance values. We then used that ATP maintenance value in our calculation of predicted growth yield for the given growth rate. This method allowed us to essentially test our model's growth yield predictions on each separate test point while training on the remaining 8 measurements. The resulting predicted growth yields are plotted in Fig. 4 along with our measured growth yields. As illustrated by this plot, our model was able to consistently predict growth yield within the 95% confidence interval of a measured test sample after being trained on a separate data set, with the exception of two predictions that were just above the 95% interval. These two small discrepancies most likely indicate random variations in these particular growth experiments that, while not particularly unusual, cause our linear model to slightly overestimate growth yield.

We also used the full data set of growth rates and methane evolution rates to set final values for growth-associated maintenance (GAM) and non-growth-associated maintenance (NGAM). The GAM represents how much ATP hydrolysis is required to support growth-related processes, and NGAM represents how much ATP hydrolysis is required for non-growth-associated cellular upkeep. GAM was originally set as 40.11 mmol per g of cell mass, a relatively low value compared with that of a fast-growing bacterial species; for example, the GAM for Escherichia coli is 59.81 mmol per g of cell mass (66). NGAM, represented by simple ATP hydrolysis, was unbounded in our first draft reconstruction and took on a value of 0 during all model simulations. After training on our full data set, we set our GAM and NGAM values to 169.9 mmol ATP per g of cell mass and 5.0 mmol ATP per gram of cell mass h^−1^, respectively (see Text S2 in the supplemental material). Notably, these maintenance values are much higher than those in other methanogen models; for example, the fellow methanogen Methanosarcina barkeri was reported to have a GAM of 65.00 mmol per g of cell mass (55), about 38% of our calculated value. This difference is reflective of the observed differences in growth yield for these organisms during growth on H_2_ and CO_2_. Using the same formula for growth yield in each case at nearly identical doubling times of 12 h, M. maripaludis grew at a yield of about 33% of that reported for M. barkeri (55). Thus, although we calculated unusually high ATP maintenance requirements for growth, these high values reflect observed differences in growth data compared to a methylotrophic methanogen growing on the same substrates.

### Gene knockout validation.

Gene knockout experiments present a different method for validating a metabolic reconstruction. At its core, a constraint-based model is built around standard gene-protein-reaction relationships that connect genotype to growth phenotype. Thus, comparing model predictions of gene knockout lethality provides an excellent way to quantitatively test the model and also potentially discover metabolic/genetic flexibility in M. maripaludis. This process hinges on the availability of gene knockout data for the organism being modeled. Unlike a traditional model organism, such as E. coli (67), M. maripaludis lacks this abundance of *in vivo* gene knockout data; however, it has been used for transposon mutagenesis to calculate an essentiality index of all of its genes (68). Although this data set does not contain the same quality of knockout data as actual knockout experiments, the essentiality index provides a valuable first-pass test set for gene essentiality of our model. Results of comparing our model's predictions to this data set can be found in Text S1 in the supplemental material.

**FIG 5:**
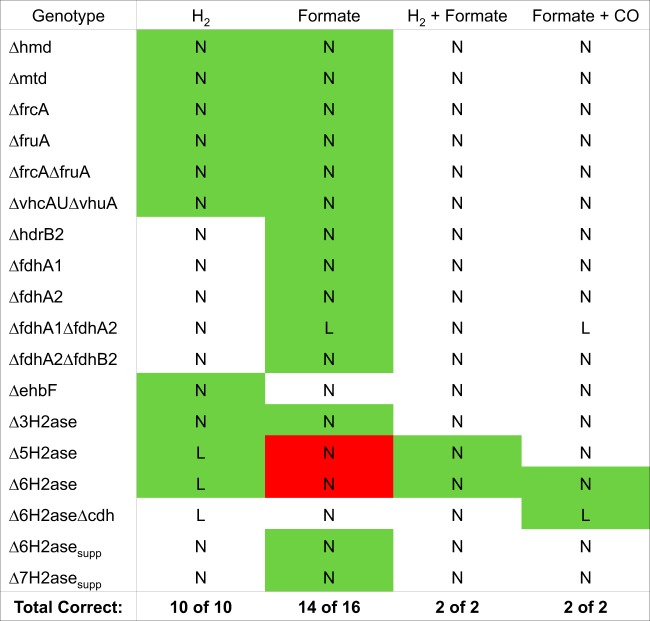
Knockout lethality predictions from running FBA on our models show close agreement with experimental results of hydrogenase knockouts. Green boxes indicate growth phenotypes where our models correctly replicated experimental results, red boxes indicate growth phenotypes where our models were incorrect, and white boxes indicate growth phenotypes where we lacked experimental validation data. Across the full spectrum of conditions, our models correctly predicted 28 of 30 conditions (93%), resulting in a strong Matthews correlation coefficient of 0.78. This suggests that our reconstruction produces models that accurately depict the effects of genotype alterations on growth phenotypes. L, lethal prediction; N, nonlethal prediction.

The bulk of available gene knockout data involves hydrogenase knockouts under different growth conditions. For our test set, we assembled a knockout panel of 30 binary growth phenotypes from previous work (42–47). Although the breadth of these knockout genotypes is limited, they are involved in various important portions of the methanogenic pathway; therefore, they provide a good idea of how well our model can predict knockouts in central catabolism. In comparison to these data, as shown in Fig. 5, our model achieved 93% prediction accuracy and an MCC of 0.78. This result was particularly encouraging because we avoided training our model on this data set in the interest of preventing overfitting our model to the validation set.

As shown in Fig. 5, our model incorrectly predicts knockout lethality for 2 cases; both of these incorrect predictions have similar bases in the model. In these cases, knockouts of 5 or 6 hydrogenases are experimentally found to be lethal in formate-grown cells, yet our model predicts these knockouts are nonlethal. The reason for this disagreement lies in our innate assumption that every reaction performs at 100% efficiency, an ideal scenario that is not achievable in an actual organism. Methanogenesis cannot be expected to operate at 100% enzyme efficiency, as some substrates and electron carriers will not react; thus, it can be considered a leaky process where a portion of the metabolites are unused in every cycle. Specifically, in the Δ5H_2_ase and Δ6H_2_ase knockouts (Fig. 5), small amounts of hydrogen are synthesized in biosynthetic reactions. Eha hydrogenase remains active in each mutant and can use H_2_ to supply anaplerotic reduced ferredoxin for methanogenesis. However, in reality, an additional nonstoichiometric amount of hydrogen is required (43). Thus, the actual mutants cannot grow on formate alone and require hydrogen.

Notably, most of our knockout predictions were made with glyceraldehyde-3-phosphate ferredoxin oxidoreductase (GAPOR) constrained to carry zero flux. The GAPOR reaction is ferredoxin reducing and can serve as a supplemental source of reduced ferredoxin for growth on formate in the case of Eha knockout (45). However, in wild-type strains the expression of GAPOR is not sufficient to support growth in the absence of other hydrogenases (e.g., the Δ5H_2_ase and Δ6H_2_ase mutants). As demonstrated previously, a mutation resulting in overexpression of the GAPOR operon allows for growth of these mutants (Δ6H_2_ase_supp_ and Δ7H_2_ase_sup_) on formate (45). To best reflect these genotypic differences, we altered the bounds of the GAPOR reaction (rxn07191[c0]) in our knockout simulation script, constraining the reaction to zero flux in all cases except those of the Δ6H_2_ase_supp_ and Δ7H_2_ase_sup_ mutants. This introduces the variables of overexpression mutations and ferredoxin promiscuity, which creates new links between pathways not normally occurring in the wild-type strain.

## DISCUSSION

Genome scale metabolic reconstructions provide a wide lens for studying biochemical complexity in a computational setting. We used likelihood-based gap filling and meticulous manual curation to build iMR539, a comprehensive reconstruction of M. maripaludis that incorporates electron bifurcation to portray cyclical hydrogenotrophic methanogenesis. We incorporated many unique pathways that differentiate our network from those for other organisms, creating a novel tool for understanding and probing more deeply into hydrogenotrophic methanogenesis. The resulting network model compared favorably with measured growth yield and gene knockout data and provided a platform to develop a new method for estimating overall free-energy generation during steady-state growth.

The energetic coupling of heterodisulfide reduction to ferredoxin reduction for the first step of cyclical methanogenesis is in stark contrast to existing methanogen models that contain the linear one based on oxidative (electron transport) phosphorylation (27, 55, 56). While the linear model is correct for methanogens with cytochromes, it is not correct for methanogens without cytochromes, such as M. maripaludis. We have demonstrated that, in the absence of a membrane-bound HdrDE complex, this coupling via electron bifurcation is essential for predicting growth in our network. Furthermore, constraining the energy-conserving Eha/Ehb reaction to a minor metabolic role provides a stoichiometric hypothesis for the inability of M. maripaludis to grow aceticlastically and will undoubtedly influence model predictions moving forward.

Although analysis of various pathways seems to suggest ferredoxin specificity from knockout data or from inclusion of a ferredoxin gene(s) within related enzyme operons (71), other data indicate some limited flexibility in ferredoxin cross usage (45, 69). Additionally, many *in vitro* enzyme assays studying electron bifurcation in methanogens utilize clostridial ferredoxins (11). M. maripaludis has 59 annotated iron sulfur proteins (13), of which there are at least 16 known ferredoxins (68), opening multiple possibilities of ferredoxin cross usage. Ferredoxin specificity/promiscuity for certain reactions remains an open question that could profoundly affect electron carrier utilization and have implications in native and mutant genotypes, a possibility we have acknowledged by allowing either promiscuous or specific ferredoxins in our reconstruction (see Text S4 in the supplemental material). Using this function theoretically tightens the coupling between the aforementioned reactions by restricting each set to one pool of electron carriers; however, this change currently has minimal effects on predicted growth yields and fluxes. The difficulty of implementing ferredoxin specificity in iMR539 illustrates a need for future studies to demystify the roles of different ferredoxin species for M. maripaludis metabolism, particularly in electron bifurcation. A clearer picture of ferredoxin promiscuity could notably impact predicted flux distributions and gene knockout phenotypes and have important implications for hypothesizing strain designs; thus, including multiple ferredoxins could be vital for effective metabolic engineering.

Beyond electron bifurcation itself, we added numerous uncommon biosynthetic pathways to our network from literature sources that further separate it from models of other organisms. These pathways included syntheses for methanogenic coenzymes, archaellin sugars, and archaeol lipids, as well as a relatively novel sulfur assimilation pathway. Additionally, using likelihood-based gap filling helped us automatically identify 66 more genes, increasing the gene coverage of our reconstruction prior to the start of manual curation and assigning reaction likelihood scores for many reactions that lend a measure of confidence to the network. These modifications demonstrated the need for rigorous manual curation to add known biochemical pathways that were not part of the automated reconstruction and remove pathways that are known not to function in the organism. By employing these methods and by working collaboratively with various groups intimately familiar with M. maripaludis biology, we have created a reconstruction that maximizes consistency with biochemical literature for our organism. The efficacy of these methods is shown not only in the qualitative accuracy of our reconstruction but also in the formidable quantitative capabilities of the resulting model. Although growth yield validation is not an absolute measure of model performance, our model's ability to closely reproduce experimental results in an LOOCV setting that mitigated overfitting suggested a high propensity for generating viable growth predictions. Moreover, the relative consistency between measured and predicted values indicated our model's robustness for predicting growth yields across a range of different dilution and methane secretion rates. Furthermore, our model's comparatively high correlation (MCC of 0.78) with experimental knockouts suggested that our model is an excellent predictor of growth phenotype based on genotype changes in central carbon metabolism. For context, this MCC compares favorably with the single-gene-deletion overall MCCs for Saccharomyces cerevisiae models, among the best curated and most revised models to date, that range from 0.38 to 0.63 (70).

For a methanogen living close to the edge of thermodynamic feasibility, we also thought it salient to include some calculation of overall free energy when simulating our model. Thus, we have introduced a novel method of predicting overall model free-energy generation based solely on standard free energies and concentrations of exchange metabolites. This is in contrast to existing methods that predict reaction by reaction directionality using free energies and concentration ranges for all metabolites; our implementation is on a much smaller scale and is aimed at approximating the overall state of the system rather than constraining individual reactions. We expect that this straightforward calculation (see Materials and Methods) will be a useful addition to our model, particularly as we aim to use it as a platform for generating possible strain designs. With regard to free energy, methanogens are particularly notable in that they subsist close to the thermodynamic limit to support growth (30). It follows that for any potential strain design, we must pay particular attention to the overall free energy of our system, lest it dip below this vital threshold. It may also provide a metric for differentiating between multiple feasible strain designs by ranking them in order of thermodynamic feasibility. At the very least, it serves as an additional capability of our model and as a checkpoint to ensure that our overall stoichiometry matches up with overall free energy.

Although we have attempted to address as many parts of metabolism as possible, many unexplored areas of M. maripaludis metabolism still exist in our reconstruction and present ample opportunity to expand and revise our reconstruction. Our intention was to make our reconstruction decisions transparent to future users, and we hope that by providing information on the origins and likelihoods of our reactions, we can encourage exploration of as-yet-unknown pathways. In doing so, our reconstruction provides a tool to realistically portray the scope of possible metabolic capabilities of hydrogenotrophic methanogens, push forward biochemical discovery in these organisms, and unlock their potential as metabolic engineering targets.

## Supplementary Material

Supplemental material
