# Supplementary material for: Exploring Hydrogenotrophic Methanogenesis: a Genome Scale Metabolic Reconstruction of Methanococcus maripaludis
Source: J Bacteriol. 2016 Nov 18;198(24):3379–90. doi: 10.1128/JB.00571-16 (PMC5116941; doi:10.1128/JB.00571-16)
Supplement: Supplemental material [file supp_198_24_3379__index.html]

Supplemental material 

# Exploring Hydrogenotrophic Methanogenesis: a Genome Scale Metabolic Reconstruction of Methanococcus maripaludis

## Supplemental material

**Files in this Data Supplement:**

- Supplemental file 1 -

  Texts S1 (Comparison with genome scale essentiality indices), S2 (Determination of growth yield parameters), S3 (Free energy estimation capabilities), and S4 (Description of select files)

  PDF, 450K
- Supplemental file 2 -

  Data set S1 (Reactions, metabolites, genes, reference materials, and media for iMR539)

  XLSX, 201K
