# Supplementary material for: Exploring Hydrogenotrophic Methanogenesis: a Genome Scale Metabolic Reconstruction of Methanococcus maripaludis
Source: J Bacteriol. 2016 Nov 18;198(24):3379–90. doi: 10.1128/JB.00571-16 (PMC5116941; doi:10.1128/JB.00571-16)
Supplement: Supplemental material [file JB.00571-16_zjb999094267so1.pdf]

Supplementary Text S1: Comparison with Genome-Scale Essentiality Indices

A previous group performed a genome-scale analysis of gene function of *Methanococcus maripaludis* via a saturated transposon mutagenesis (TN-seq) technique on rich and minimal media (1). Although this dataset does not contain the same quality of knockout data as actual knockout experiments, it provides a valuable "first pass" test set for gene essentiality of our model. For minimal medium in particular, their data included 2 whole genome libraries of mapped insertions, each of which contained growth data for 7 (T1) and 14 generations (T2). Reasoning that essential genes would likely be conserved across mutants, they correlated number of insertions at a particular gene location with gene essentiality by calculating an "essentiality index" (EI) for each location. Based upon a set of "known essential" genes, they set a cutoff of EI ≤ 3 for essential genes, effectively creating predictions of gene essentiality for all genes.

Considering the 4 sets of library:generation combinations for minimal medium—Lib.1:T1, Lib.1:T2, Lib.2:T1, Lib.2:T2—each gene could be predicted to be essential in 0-4 cases. Rather than globally classify gene essentiality based on all 4 cases, we created 4 separate sets of essential genes by setting different essentiality thresholds. For example, in "4 instances", only genes that were predicted as essential in all 4 libraries were treated as essential genes and all other genes were considered non-essential; in "1 instance", all genes that were predicted as essential in at least 1 library were treated as essential genes. The iMR539 reconstruction shared 537 genes with this dataset, thus we were able to compare gene essentiality predictions across nearly the entire model to the TN-seq data for minimal medium.

As shown by Figure S1, different thresholds had a great effect on the EI predictions; a lower threshold necessarily caused an increase in negative (no-growth) outcomes and a decrease in positive (yes-growth) outcomes. Our model experienced no change in its gene essentiality

predictions in relation to threshold, hence a decrease in threshold resulted in improved performance on negative predictions and decreased performance on positive predictions. The threshold's effect on overall performance, displayed in Figure S2, shows that our model's predictive accuracy in the four cases ranged from 61.3-65.0% and was maximized in the "3 instances" dataset, whereas MCC ranged from 0.277-0.317 and was highest for "2 instances". This small discrepancy reflects the difference in how these metrics are calculated, with MCC putting greater emphasis on our model's improved ability to predict true negative outcomes.

Overall, this analysis revealed a slight positive correlation between EI predictions and gene essentiality predictions from out model. It is important to keep in mind that EI, like our reconstruction, is a model of gene essentiality and should not be confused for actual knockout data. Through different methods, both models provide hypotheses for gene functions outside known metabolism and could fuel future investigations to directly measure gene essentiality.

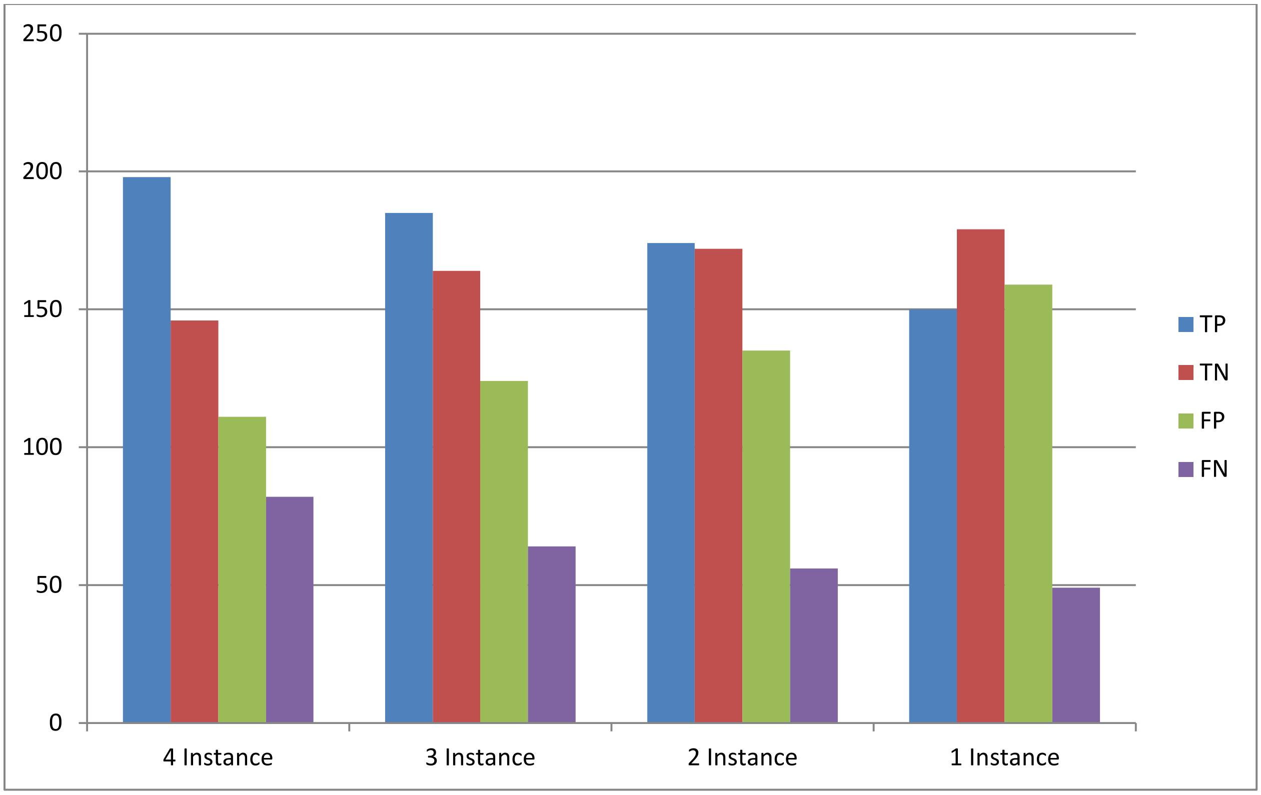

Figure S1: Comparison of model predictions with genome-scale essentiality indices (EI) on minimal media across 4 libraries. Instances indicate the threshold of libraries for qualifying a gene as lethal. Positive results indicate predicted non-lethal genes, negative results indicate predicted lethal-genes. TP: true positive, model and EI both predict non-lethality; TN: true negative, model and EI both predict lethality; FP: false positive, model predicts non-lethality, EI predicts lethality; FN: false negative, model predicts lethality, EI predicts non-lethality.

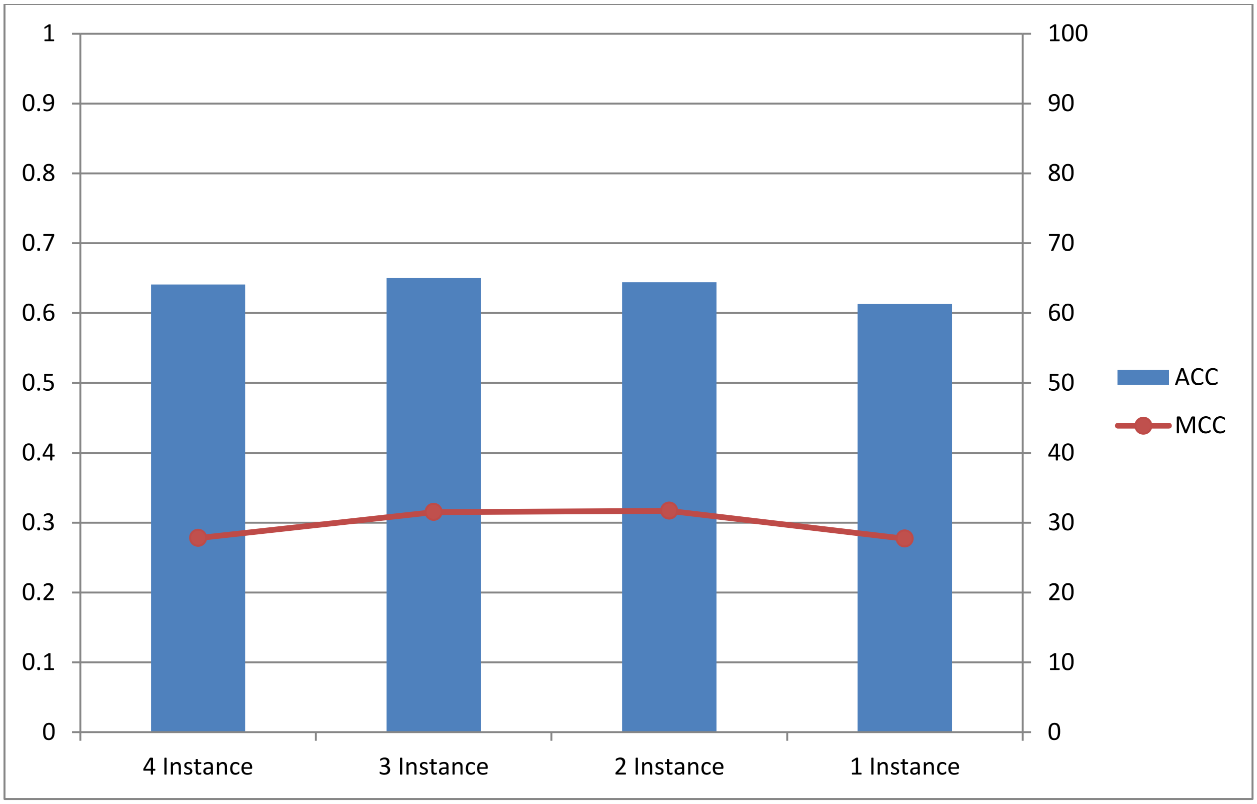

Figure S2: Matthews Correlation Coefficient (MCC; left y-axis) and predictive accuracy (ACC; right y-axis) comparing model predictions with genome-scale essentiality indices (EI) on minimal media across 4 libraries. Instances indicate the threshold of libraries for qualifying a gene as lethal.

Supplementary Text S2: Plots Illustrating Determination of Growth Yield Parameters

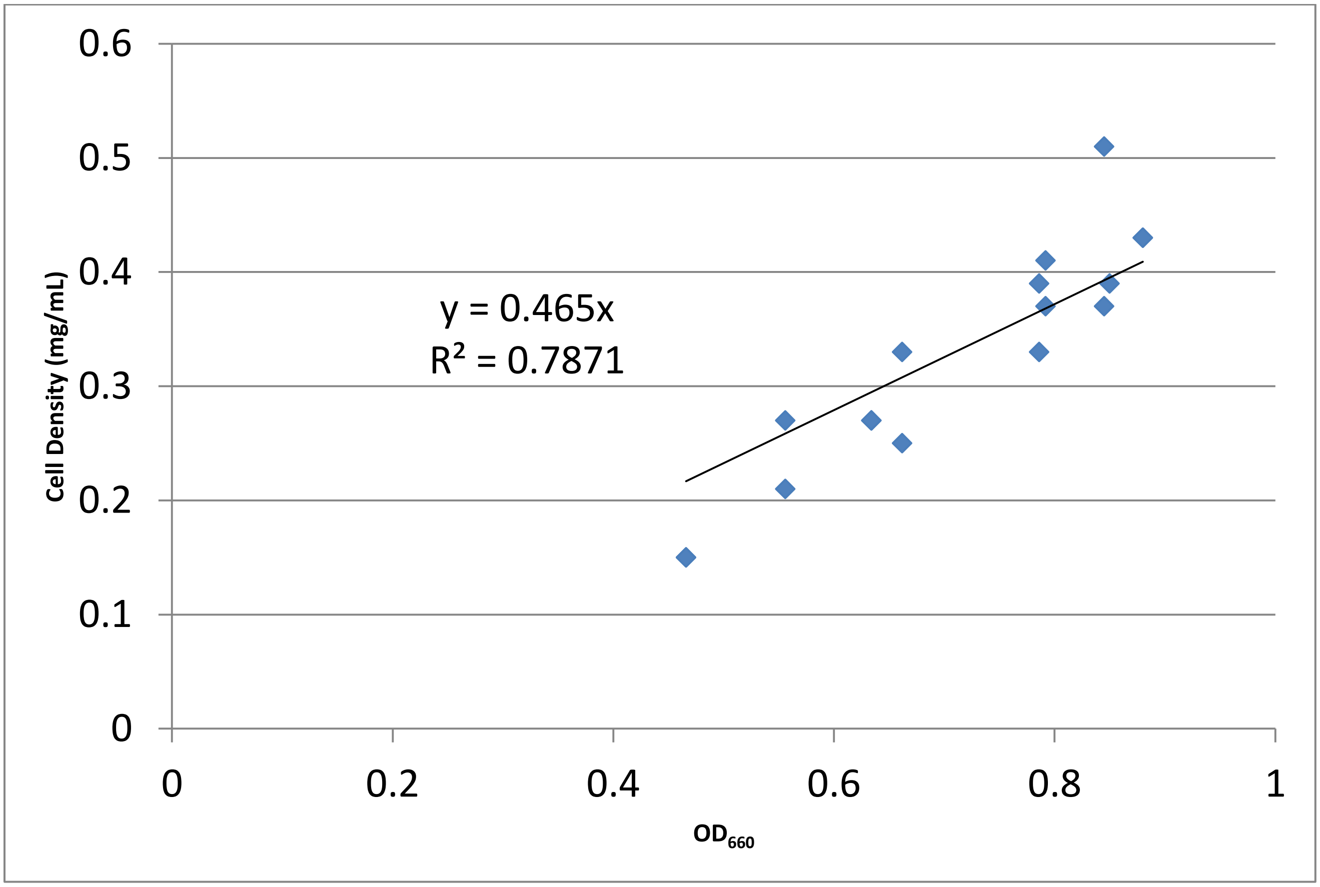

Figure S3: Determination of the relationship between cell density and optical density (OD_{660}). Linear regression was set to intersect (0,0), as cell density must necessarily be 0 when $OD_{660} = 0$. For specific methodology on how these points were gathered, see Methods.

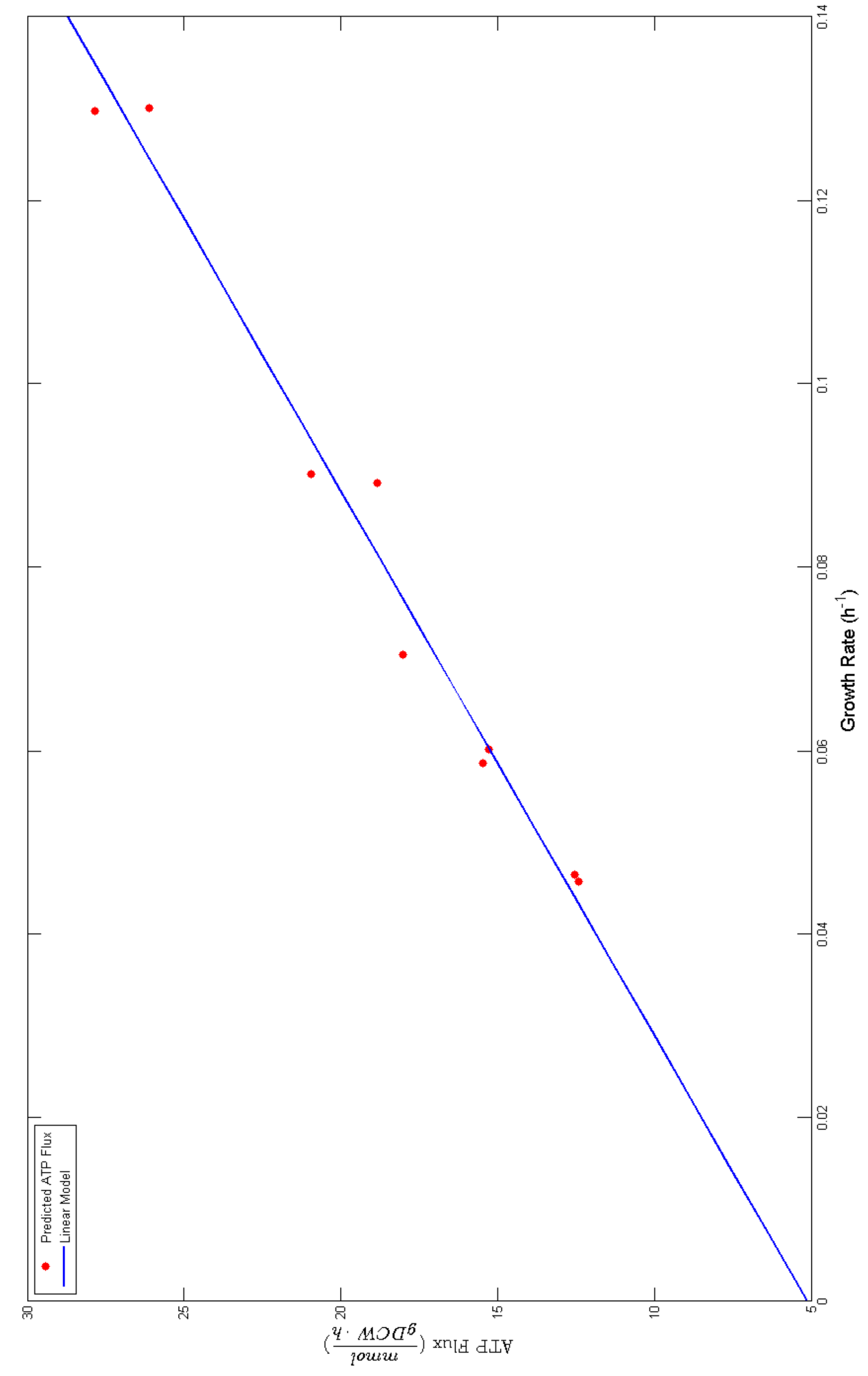

Figure S4: Illustration of the process used to determine ATP maintenance values (see Methods). Using all 9 measured samples, GAM (slope) and NGAM (y-intercept) were determined as 168.4 (mmol per grams [cell mass]) and 5.12 (mmol per grams [cell mass] per hour), respectively.

Supplementary Text S3: Demonstrating our Free Energy Estimation Capabilities

By including available free energy of formation values for exchange metabolites, we have equipped our model to quickly estimate overall free energy generation. To demonstrate this functionality, we consider the case of hydrogen concentrations in methanogenic environments. In our growth simulations we accept a list of exchange metabolites and a vector of their concentrations in units of mM (see https://github.com/marichards/methanococcus). By default, we assume that all aqueous concentrations for these metabolites are 1 mM, thus if concentrations are not supplied we calculate overall free energy as follows:

```
>> solution = maxGrowthOnH2Only(model);

Biomass flux: 0.096861

Formate flux: 0.000000
CO2 flux: -52.579347
H2 flux: -205.169846
H2O flux: 103.908440
CH4 flux: 50.000000
NH3 flux: -0.752865
PO4 flux: 0.009705
Acetate flux: 0.000000

Overall reaction:
CO2 + 4 H2 --> 2 H2O + CH4

Model overall reaction (per mole CH4)
1.05 CO2 + 4.10 H2 --> 2.08 H2O + CH4

Predicted Yield Coefficient: 2.79 gDCW/mol CH4

Expected ATP/CH4 Yield: 0.5
Predicted ATP/CH4 Yield: 0.475

Warning: All external metabolite concentrations set to 1 mM
> In maxGrowthOnH2Only at 99

Predicted Free Energy Generation: -6.457393 kJ/gDCW
```

A key feature of hydrogenotrophic methanogens is their ability to thrive in conditions with low H_2 partial pressure (~10 Pa). Converting to aqueous concentration via Henry's Law coefficient (2), 10 Pa corresponds to 7.7 x 10^{-5} mM. We can estimate overall free energy for this hydrogen concentration by specifying this parameter:

```
>> solution = maxGrowthOnH2Only(model,{'EX_cpd11640[e0]'},[7.7e-5]);

Biomass flux: 0.096861

Formate flux: 0.000000
CO2 flux: -52.579347
H2 flux: -205.169846
```

```
H2O flux: 103.908440
CH4 flux: 50.000000
NH3 flux: -0.752865
PO4 flux: 0.009705
Acetate flux: 0.000000

Overall reaction:
CO2 + 4 H2 --> 2 H2O + CH4

Model overall reaction (per mole CH4)
1.05 CO2 + 4.10 H2 --> 2.08 H2O + CH4

Predicted Yield Coefficient: 2.79 gDCW/mol CH4

Expected ATP/CH4 Yield: 0.5
Predicted ATP/CH4 Yield: 0.475

Predicted Free Energy Generation: -1.448827 kJ/gDCW
```

As illustrated by this quick calculation, we predict overall free energy of the system to still be favorable for methane generation from H_2, though of much smaller magnitude than at higher H_2 concentrations. Assuming other external metabolite concentrations remain at 1 mM, we can also conduct a short sensitivity analysis of H_2 concentration on overall free energy. Setting H_2 concentrations from (10^{-10} - 10^{0}), we can calculating the overall free energy for methanogenesis in each case produces Figure S5. This figure shows the expected logarithmic relationship between H_2 concentration and overall free energy, with $\Delta G = 0$ at $[H_2] \approx 5 \times 10^{-6}$ mM ($P_{H2} \approx 0.65$ Pa).

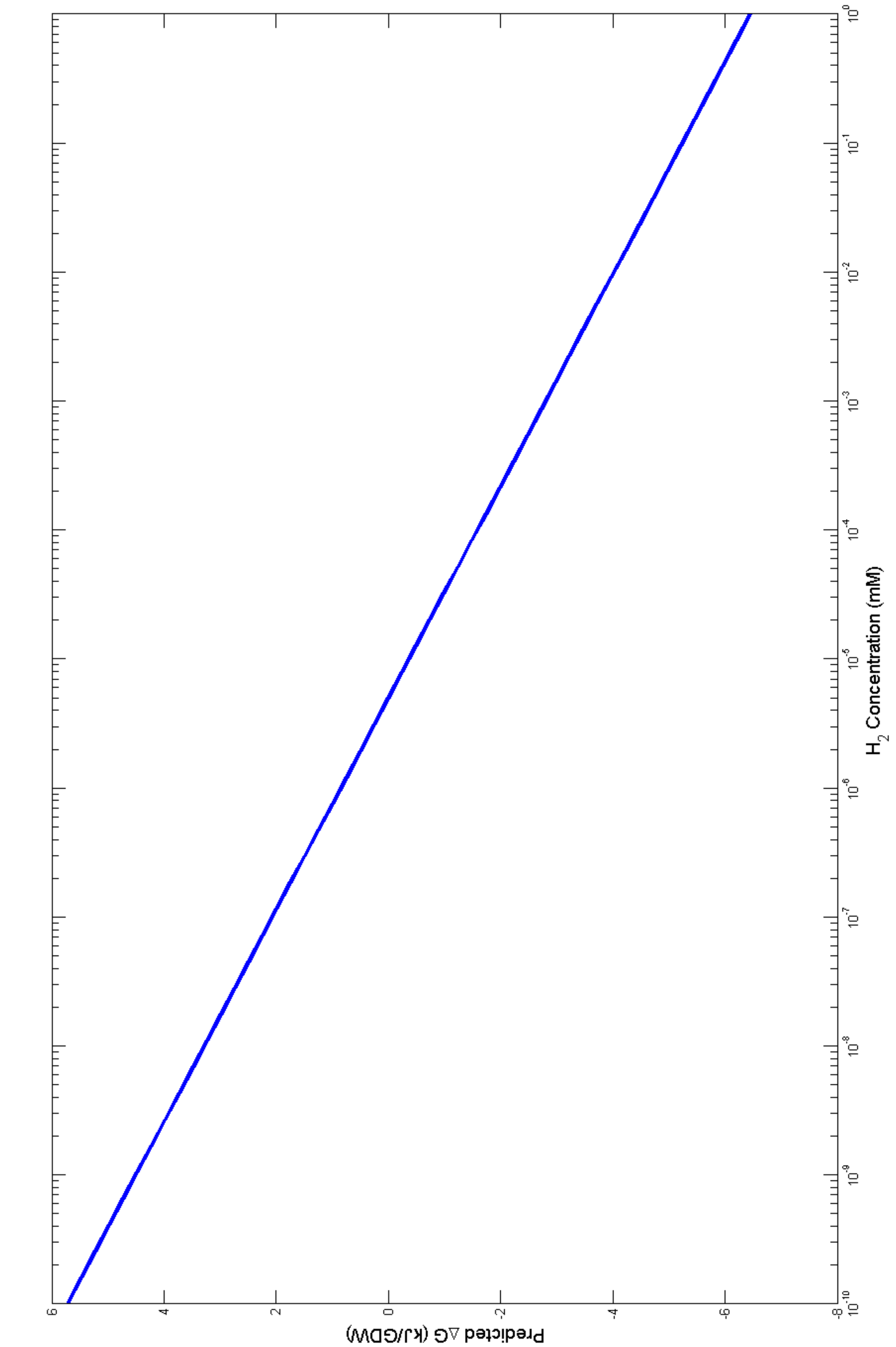

Figure S5: Semi-log plot showing our sensitivity analysis of the effects of [H_2] on overall free energy generation(ΔG).

Supplementary Text S4: Description of Select Files

We have created the iMR539 reconstruction in both XML (SBML) and Matlab data structure formats and made those available along with our scripts for working with the model. for the most up to date scripts and model files, please refer to our GitHub repository (https://github.com/marichards/methanococcus). A selection of these materials are briefly described below; please note that all scripts are dependent on the COBRA Toolbox 2.0.5 in Matlab (3)

iMR539.xml

This file contains the *M. maripaludis* model in SBML, an extensible format compatible with most constraint-based modeling platforms. It is identical to the model we deposited in the Biomodels database (4) with identifier MODEL1607200000.

iMR539.mat

This file contains the *M. maripaludis* model in Matlab data structure format, the standard format used for constraint-based modeling in the COBRA toolbox. In addition to the standard COBRA model fields, this model file includes additional metadata, including free energies of formation for exchange reactions in kJ/mmol.

maxGrowthOnH2.m

This Matlab script simulates iMR539 for maximum biomass production during growth on hydrogen with acetate supplementation and using ammonia as the nitrogen source. The script not only returns the flux solution, but also prints out physiologically-relevant parameters including relevant in/out fluxes, predicted biomass yield, and the predicted overall chemical reaction. It is dependent on the following included scripts: switchToH2.m, switchToNH3.m, setMethaneSecretion.m, optimizeThermoModel.m.

maxGrowthOnFormate.m

This Matlab script simulates iMR539 for maximum biomass production during growth on formate with acetate supplementation and using ammonia as the nitrogen source. The script not only returns the flux solution, but also prints out physiologically-relevant parameters including relevant in/out fluxes, predicted biomass yield, and the predicted overall chemical reaction. It is dependent on the following included scripts: switchToFormate.m, switchToNH3.m, setMethaneSecretion.m, optimizeThermoModel.m.

simulateKOPanel.m

This Matlab script simulates iMR539 for growth on the knockout panel shown in Figure 5 (see main text). It prints out the predicted wild type biomass production under each media formulation, followed by the predicted biomass production under each knockout condition, and

concluding with the overall predictive accuracy and Matthew's Correlation Coefficient (MCC) when compared to experimental knockout data. It is dependent on the following included scripts: switchToH2.m, switchToFormate.m, setMethaneSecretion.m.

switchToH2.m

This Matlab script changes the growth conditions for the iMR539 model such that the *in silico* media contains hydrogen and carbon dioxide as the main substrates, plus acetate as a supplementary source. It also constrains methane secretion to 50 mmol/(gDCW·h) and bounds maximum flux through Eha/Ehb to 5 mmol/(gDCW·h). It is dependent on the setMethaneSecretion.m script.

switchToFormate.m

This Matlab script changes the growth conditions for the iMR539 model such that the *in silico* media contains formate as the main substrate, plus acetate as a supplementary source. It also constrains methane secretion to 50 mmol/(gDCW·h) and bounds maximum flux magnitude through Eha/Ehb to 5 mmol/(gDCW·h). It is dependent on the setMethaneSecretion.m script.

switchToNH3.m

This Matlab script sets the nitrogen source for the iMR539 model as ammonia, the default substrate used in standard culturing conditions, by allowing unlimited ammonia uptake.

setMethaneSecretion.m

This Matlab script sets the methane secretion rate for the iMR539 model to a specified value, effectively constraining the biomass production, product secretion, and uptake rates of the model during simulation. It also constrains the magnitude of flux through Eha/Ehb to 10% of the methane secretion rate to enforce our assumption that Eha/Ehb can only play an anaplerotic role during growth.

removeEhaBounds.m

This Matlab script removes the constraints on iMR539 for the Eha/Ehb reaction(s), allowing flux up to standard lower/upper bounds of -1000/1000.

optimizeThermoModel.m

This Matlab script adds overall free energy predictions for simulating the iMR539 model, returning a predicted value for overall Gibbs free energy. It is dependent on the presence of a "freeEnergy" array in the supplied model, a feature which exists only in the Matlab version of iMR539 (iMR539.mat). For more information on this script, please refer to Supplementary File 4.

switchToSpecificFd.m

This Matlab script changes several reactions in the iMR539 model by creating 2 new specific types of ferredoxin. The original ferredoxin species—cpd11620 and cpd11621—are considered "promiscuous" ferredoxins and this script creates specific species—Fdrd*1/2 and Fdox*1/2—that each appear in only a few reactions. These changes have little effect on standard growth predictions; however, they may prove important in future simulations involving ferredoxin specificity.

References

1. **Sarmiento F**, **Mrázek J**, **Whitman WB**. 2013. Genome-scale analysis of gene function in the hydrogenotrophic methanogenic archaeon *Methanococcus maripaludis*. Proc Natl Acad Sci **110**:4726–4731.

2. **Sander R**. 2015. Compilation of Henry's law constants (version 4.0) for water as solvent. Atmos Chem Phys **15**:4399–4981.

3. **Schellenberger J**, **Que R**, **Fleming RMT**, **Thiele I**, **Orth JD**, **Feist AM**, **Zielinski DC**, **Bordbar A**, **Lewis NE**, **Rahmanian S**, **Kang J**, **Hyduke DR**, **Palsson BØ**. 2011. Quantitative prediction of cellular metabolism with constraint-based models: the COBRA Toolbox v2.0. Nat Protoc **6**:1290–1307.

4. **Li C**, **Donizelli M**, **Rodriguez N**, **Dharuri H**, **Endler L**, **Chelliah V**, **Li L**, **He E**, **Henry A**, **Stefan MI**, **Snoep JL**, **Hucka M**, **Novère NL**, **Laibe C**. 2010. BioModels Database: An enhanced, curated and annotated resource for published quantitative kinetic models. BMC Syst Biol **4**:92.
